# Developing a facilitation model to promote organisational development in primary care practices

**DOI:** 10.1186/1471-2296-7-38

**Published:** 2006-06-19

**Authors:** Melody Rhydderch, Adrian Edwards, Martin Marshall, Glyn Elwyn, Richard Grol

**Affiliations:** 1 Centre For Health Sciences Research, Neuadd Meirionydd, Heath Park, CF14 4YS, Cardiff, UK; 2 Department of Health, Richmond House, 79 Whitehall, London, SW1A 2NS, UK; 3 Centre For Quality of Care Research, University of Nijmegen, 6500 HB, Nijmegen, The Netherlands

## Abstract

**Background:**

The relationship between effective organisation of general practices and health improvement is widely accepted. The Maturity Matrix is an instrument designed to assess organisational development in general practice settings and to stimulate quality improvement. It is undertaken by a practice team with the aid of a facilitator. There is a tradition in the primary care systems in many countries of using practice visitors to educate practice teams about how to improve. However the role of practice visitors as facilitators who enable teams to plan practice-led organisational development using quality improvement instruments is less well understood. The objectives of the study were to develop and explore a facilitation model to support practice teams in stimulating organisational development using a quality improvement instrument called the Maturity Matrix. A qualitative study based on transcript analysis was adopted.

**Method:**

A model of facilitation was constructed based on a review of relevant literature. Audio tapes of Maturity Matrix assessment sessions with general practices were transcribed and facilitator skills were compared to the model. The sample consisted of two facilitators working with twelve general practices based in UK primary care.

**Results:**

The facilitation model suggested that four areas describing eighteen skills were important. The four areas are structuring the session, obtaining consensus, handling group dynamics and enabling team learning. Facilitators effectively employed skills associated with the first three areas, but less able to consistently stimulate team learning.

**Conclusion:**

This study suggests that facilitators need careful preparation for their role and practices need protected time in order to make best use of practice-led quality improvement instruments. The role of practice visitor as a facilitator is becoming important as the need to engender ownership of the quality improvement process by practices increases.

## Background

Emerging evidence about the link between organisation and quality of care has resulted in interest in stimulating primary care practices to assess and improve their levels of organisation [1-6]. This is designed to complement existing approaches which encourage practices to undertake audits, implement guidelines and change relevant working practices, often with the help of a visitor who plays an educational or 'expert' role with the practice [7,8]. However, the role and method for such a visitor to stimulate practice-led quality improvement are not well understood.

Previous reviews of organisational assessments and the theory underpinning them suggests that the dominant approach is that of professionally-led accreditation schemes [9,10]. This is based in large part on systems theory which predicts that standard setting, data measurement and feedback are triggers for improvement. In this context, the role of the practice visitor is primarily that of assessor, their job being to visit the practice, conduct an assessment against external standards and arrange feedback to the practice.

However, an alternative approach to assessment is to work alongside a practice team enabling them to focus practically on the process of change, helping them to identify where they are now, where they would like to be and how they would like to get there [11]. It is based upon organisational development theory which assumes that change can be planned and that its effectiveness depends upon overlap between individual and organisational goals; change has to be seen to be in everyone's interest [12-16]. Combining facilitation with the use of a prescriptive process for change is considered one mechanism for change by organisational development theory. The Maturity Matrix is a validated quality improvement instrument based upon organisational development theory and designed to be used by general practice teams with the aid of a facilitator to assess their existing levels of organisational development and to plan quality improvements [11]. An overview of the Maturity Matrix instrument and the role of the facilitator are described in Table 1. Eleven areas of organisation known as dimensions are described by the Maturity Matrix. Each dimension consists of eight descriptions of activity that together describe incremental progress from basic to more developed arrangements.

**Table 1:** The Maturity Matrix and the role of the facilitator: An overview

Eleven areas, known as dimensions, are covered by the Maturity Matrix and these are listed below. Each dimension consists of eight stages that describe a progression from very basic practice to more developed arrangements. For example, the first dimension, clinical data, describes how practices typically progress from having paper based systems to having computer based systems capable of storing and analysing information about prescribing, referrals and diagnostic coding.	
Dimension	Description: Organisational activities that:

1. Clinical data	describe the development of a clinical records system.
2. Audit of clinical performance	that support the practice in undertaking audit activity.
3. Use of guidelines	describe the way that a practice uses clinical guidelines.
4. Clinician access to clinical information	ensure that health professionals have access to clinical information.
5. Prescribing	support the proactive use of prescribing data as a mechanism for quality improvement and cost containment.
6. Human resources	ensure attention to policies and systems to support staff management.
7. Continuing professional development	ensure education and training for health professionals and other practice staff is based on an organisational development plan.
8. Risk management	support the identification, analysis and management of clinical and non clinical risk.
9. Practice meetings	enable effective team meetings.
10. Sharing information with patients	support patients being given information that is evidence-based and tailored to their personal needs and contexts.
11. Learning from patients	recognise patients as an important source of feedback on the organisation of services and performance of the providers and the organisation.

Facilitator role and training

The facilitator liaises with the practice to arrange for as many members of the practice team as possible to be present. A session typically lasts 1 to 1.5 hrs. The facilitator introduces the Maturity Matrix, talks about the process and takes any questions or comments. They then give a copy of the instrument to each member of the practice team and ask them to complete the Maturity Matrix individually. It takes approximately ten minutes for participants to decide where they think their practice is with regard to each of the eleven dimensions. The facilitator then initiates a discussion about each dimension in turn, encouraging participants to move from individual perspectives to reach a team consensus about the practices existing levels of organisational development and how they would like to improve. At the end of the session, the facilitator summarises the main points and agrees the next steps with the practice. Facilitators attend a standardised training programme combining didactic input about the Maturity Matrix with simulated practice using role plays, video feedback and facilitated discussion.

To facilitate means 'to make possible, aid and give scope'[17]. Facilitation is also described as 'the provision of opportunity, resources, encouragement and support for the group to succeed in achieving its own objectives and to do this through enabling the group to take control and responsibility for the way they proceed' [18,19]. Basic generic facilitation skills include: listening, questioning, encouraging participation, checking meaning, challenging, reflection, and summarising [17,20,21]. However, we are not aware of any previous work undertaken to examine how facilitation skills can best be combined to stimulate organisational development in general practices using an instrument that describes the start point, process and end point [9].

The aim of this study is to explore how facilitators support practice teams in identifying areas for growth and development using the Maturity Matrix. The first objective for the study was to identify elements that comprise an effective model of facilitation skills for the Maturity Matrix assessment process. The second objective was to explore how and to what extent the facilitators employed the skills described by the model and its elements. However, 'organisational development' and the 'Maturity Matrix' are essentially organising frameworks (a theory and a tool respectively), applied to create a structure to help practices focus their efforts on making quality improvements. A wider potential message exists as a result of understanding the way in which facilitators work with practices using the Maturity Matrix. Practice visitors may seek to expand their repertoire of skills to include facilitation instead of (or sometimes as well as) education and assessment. For this to be achieved, it is important to ask how facilitation skills differ from those used in assessment and education, and how and when they can best be deployed. The results of this study will contribute to the more effective preparation of facilitators to use the Maturity Matrix and help practice visitors to be more aware of the variety of roles which they can adopt to help stimulate quality improvements.

## Methods

This study forms part of a wider evaluation of the Maturity Matrix for which ethical approval was obtained via the Wales Multi-centre Research Ethics Committee in May 2003.

### Design

A qualitative study design was adopted, consisting of the analysis of transcripts of audio taped Maturity Matrix sessions. A model describing effective facilitation of the Maturity Matrix was developed by reviewing the literature on facilitator skills relevant to quality improvement in primary care. The model specifies areas and skills within each area against which the data extracted from the transcripts could then be evaluated.

### Sample and data collection

Data were collected from a convenience sample of UK practices (n = 26) who took part in Maturity Matrix sessions as part of a feasibility study on the use of the Maturity Matrix in European primary health care settings [22]. The practices were based in Wales and the North of England. The data for this study were collected between January and April 2004 using sessions led by two trained facilitators. Every practice that was invited to take part in the study accepted and no practices dropped out having agreed to take part.

The facilitators were both experienced in facilitating general practice teams as part of other quality improvement and research projects for an academic department of primary care, and were considered to be competent for their role. This is an important contextual issue because facilitator competence is not being evaluated in this study, but rather the focus is on the facilitation process and the opportunity to use the skills proposed by the model proposed below.

Each Maturity Matrix session was audio taped. The practices were stratified according to size. Twelve tapes were chosen to be fully transcribed with the intention of selecting tapes representing three practice size categories. The selection of tapes was made with the aim of reflecting approximately half of the sample within each size category. The transcribed sample consisted of two single-handed practices, four two-partner practices and six group practices consisting of three or more partners.

### Identifying effective facilitator skills from literature and transcripts

Elwyn, Greenhalgh and Macfarlane describe facilitator skills relevant to running small group sessions in a healthcare context [20]. These include using open and closed questions, probing, eye-contact, echoing, checking and formulating meaning. Teurfs and Gerard identify four essential facilitator skills that reflect values espoused by Schein's work on process consultation: inquiry and reflection, listening, suspension of judgment and assumption identification [23]. Heron suggests that the skills adopted by a facilitator at any given time will be influenced by the dimension in which a group is working [24]. He describes six dimensions that describe group activity: planning, meaning, confronting, feeling, structuring and valuing and three styles of facilitation: hierarchy, co-operation and autonomy. Duffy and Griffin's work on facilitator skills for primary care teams suggests that skills and qualities include: flexible style, respect for others, honesty, neutrality, knowledge of context and process, enthusiasm and conflict management [19].

### Constructing a model describing good practice: developing areas

**Figure 1:**
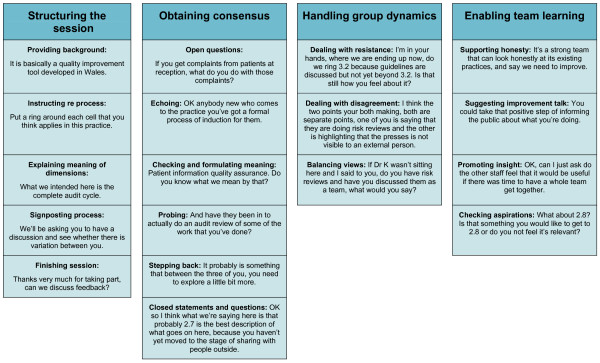


The model is illustrated in Figure 1. It contains four areas, each area describing facilitator skills associated with its use. The first area, 'structuring the session', describes the skills required by the facilitator to administer the Maturity Matrix using a standard process and consists of five skills: *providing background*, *instructing*, *explaining meaning*, *signposting process *and *finishing the session*. There is a timing factor present such that the first skills listed are more important at the beginning of the session and the last skills listed are important towards the end of the session. The second area, 'obtaining consensus', describes facilitator skills needed to enable teams to assess their existing levels of organisational development using the Maturity Matrix. Six skills are described: *open questions*, *echoing*, *checking and formulating meaning, probing, stepping back *and *closed statements and questions*. As with the first area 'structuring the session', a timing factor exists in the model, whereby the first skills listed are more important early on the discussion about each dimension and the last skills listed become more important as the discussion about each dimension progresses. Thus these six skills need to be used as part of an iterative cycle as each of the eleven dimensions is discussed.

The third and fourth areas refer to higher order facilitator skills likely to be possessed by more experienced facilitators. The third area, 'handling group dynamics', requires the facilitator to be able to deal with potentially difficult situations such as *resistance to the process*, *disagreements *amongst team members and *balancing views*. The fourth area, 'enabling team learning', describes four facilitator skills that enable the practice team to move from a position of assessing existing levels of organisational development to planning improvements to the organisation of their practice. These are: *supporting honesty*, *suggesting improvements*, *promoting insights *and *checking aspirations*.

### Data extraction and analysis

MR and AE considered four transcripts independently for evidence of whether further skills or areas should be included within the model before the remaining transcripts were analysed. A coding template was created to reflect the structure of the model and each transcript was systematically reviewed and text coded according to the model by MR. This structured approach is recommended by Miles and Huberman (1984) whereby a set of codes is derived from existing literature and these are then checked by a preliminary reading of some of the data [25,26]. The codes were used as data management tools and segments of text were categorised according to the coding template. Each of the twelve transcripts was compared to the four areas and subsets of skills and examples of effective and less effective facilitator practices were extracted and assimilated using Atlas.ti software [27]. MR and AE met regularly throughout the coding process to review coding decisions.

## Results

Data were examined and analysed for examples of facilitator behaviour that could be classified using the model described in Figure 1. Figure 1 provides one example of effective facilitator behaviour for each of the skills.

With regard to the robustness of the facilitation model constructed to describe facilitation using quality improvement instruments, the data extracted from the transcripts could be effectively categorised according to the model.

### Area 1: Structuring the session

Overall, the facilitators managed each Maturity Matrix session in accordance with their training by *providing background information*, *instructing*, *explaining the meaning of dimensions*, *signposting process *and *finishing the session *. The quote below provides an example of a general introduction to a Maturity Matrix session. This basic skill set is important if the Maturity Matrix sessions are to be administered in a standardised way. The distribution of the data across the five skills in this area, suggests that three of the skills, *providing background*, *instructing *and *finishing the session *describe issues that the facilitators only needed to use once in each session. The two other skills, *signposting process *and *explaining the meaning *of dimensions were frequently used throughout the session by the facilitator to provide structure and appropriate process for the participants.

*F: Thank you for taking part in this study. What I'd like you to do now please is to look at the form in front of you, which contains the Maturity Matrix. This is a quality improvement tool and it is not designed to be a quality assessment so we're not saying that, you know, your practice is at 1.5 on dimension 1 so you are better than other practices. It's simply for practice staff within practices to reflect on the services that they provide. What I'd like you to do is to go through the 11 different dimensions and for each of the cells in the columns for those dimensions, to put a circle around those cells which you think are applicable to this practice. For number one for instance, clinical data where it says No. 3 registration and repeat prescribing on computer, if you think that applies in this practice please put a circle around it; and in any cell in any dimension you don't think applies to this practice, please leave blank. Then what we'll do, we'll have a discussion to see what level of agreement there is within the practice about which cells are met and which aren't. So if I could ask you please to spend about 10 minutes or so filling out each of the dimensions*.

Where facilitators were under time pressure, they were not easily able to take the time to open the discussion about particular dimensions, by widely inviting participants to comment. The result was a very brief dialogue, where the emphasis was on agreeing a practice 'score', rather than on discussing potential improvements. In these instances, the facilitators did not probe or encourage the participants to discuss how their practice had developed with regard to use of audit.

### Area 2: Obtaining consensus

The facilitators appropriately used open questions to initiate discussions about each of the 11 dimensions covered by the Maturity Matrix. The use of *open questions *was the most frequently used skill. When an open question had been asked, members of the group offered their views about the development of their practice. During this process, the facilitators used the skills of *echoing*, *checking *and *formulating meaning *to expand the discussion. The quote below contains an example, where the facilitator is working with dialogue from two participants coming at the topic of clinical audit from two different perspectives. Once a variety of views were put forward, the facilitators began to ask more *probing *questions, usually focusing on one or two areas within a dimension, where the group was trying to reach a consensus view. With some practices, the facilitators were able to *step back *and the group members would discuss their organisational development with each other. When practice teams were able to debate amongst themselves, this sometimes led to a discussion about where and how to improve, with facilitators adopting the skills associated with area 4: team learning.

F: Ok, would you like to explain why you thought 2.2 was appropriate?

*P1: Well I don't particularly work in this area, so it's not that I actually know. I just sort of get the general feeling that we're doing lots of data collection but not always doing a complete audit cycle. Maybe that's just because I don't get the information back. It's like you give information on something to others doing an audit, but then maybe there's not been any feedback as to whether it made a difference*.

*F: So it's not fully utilised and examined and, if you like, lessons drawn from that is what you're saying, for some areas of audit*.

*P2:I was going to ring that but then I thought that sometimes we do audits for say, the primary care trust, but it's for their benefit, so we don't see the full circle and sometimes it's not always to benefit us*.

F: So it's not fed back to you in a way that you can say this is what we need to do to get better and then review it later; so it's not the full cycle but for one or two conditions, looking at 2.4 for example, we say regular audit cycles completed for one or two chronic conditions, would that be true?

*P1:Yeah*.

P2:I'd say more than that

F: So you'd say more?

Although, the use of *closed statements and questions *is one of the skills described by the model in figure 1, the frequency with which it was found was more limited than the use of *open questions*. Given that 12 practices each discussed 11 dimensions, many more examples could have been found. Instead, facilitators were less effective at summarising and confirming the group consensus about each dimension. There were also examples where closed statements and questions were used prematurely in the discussion about each dimension, leading to little potential for group discussion and team learning.

### Area 3: Handling group dynamics

Data were extracted relating to the facilitators' skills at handling group dynamics. The facilitators did not use these skills with approximately half of the practices in the sample, suggesting that some practices have a more straightforward dynamic in which discussions occur. The facilitator skill called *dealing with resistance *is about communicating with an individual who seems to be resisting the process or content, over and above debating the development of the practice. The quote below shows the facilitator carefully reframing the phrase 'risk review' to make it practical and relevant to the participant without becoming involved in verbal sparring.

P1: What is a risk review?

F: What did you interpret a risk review as?

*P1: Well I interpreted a risk review as what they do at the PCT (Primary Care Trust), which we don't even remotely do, so I didn't tick any of these*.

*F: But it could be as simple as undertaking a risk review of the reception area from a health and safety perspective*.

*P1: Which, as you know, is something you can't do unless you've done training, been on all sorts of management training courses and things like that, you know, risk is an in-thing at the moment and there are people specialising in this and earning lots of money carrying out risk reviews*.

F: If you don't take it at that very sophisticated level but you look at it in the terms of something Sue and I discussed this morning where there was an incident where patients of the same name were confused, I mean that could be part of a risk review because that could apply more widely than that one episode couldn't it?

The other two skills in this area, *dealing with disagreement *and *balancing views *describe facilitator behaviours designed to ensure that the discussion reflects a range of views. Where disagreement between participants occurred, the facilitator interjected to find common ground through questioning, (see quote in Figure 1 under this area). Facilitators were particularly sensitive to the views of reception staff whose perspectives were often overlooked by the doctors and nursing staff. However they sometimes struggled when handling the group dynamics where there was ongoing disagreement between participants across dimensions or where one individual was resistant to some of the dimensions covered by the Maturity Matrix.

### Area 4: Enabling team learning

This area describes four skills. The first skill, *improvement talk*, describes the moment where the participants begin to discuss improvements that they could make to the organisational arrangements in their practice. It was usually the result of the facilitator prompting them to discuss current progress and to think about their aspirations. The quote below contains an example of a practice team talking about making improvements to their patient leaflets.

*F: OK I would just make comment maybe on that because one of my checklists is looking at leaflets and you don't actually have many health leaflets in your waiting rooms. You've got general kinds of leaflets but you don't have diabetes information*.

*P1: We need an update of our leaflets from health promotion actually*.

*F: You haven't got much space either I know, so....*.

*P2: We have leaflets with the practice nurse, things that are given out at clinics*.

*P1: Let's update the leaflets. I agree we are short. I was looking the other day and we're a bit short of the health ones at the moment; we need to touch them up a bit*.

*F: If you take the view that they are useful for patients who may not think they have a problem but while they are waiting they'll look at something and then they'll think, oh yeah that applies*.

However, the facilitator was not always able to encourage participants to move beyond assessing their current levels of organisational development to planning improvements. This is potentially an area where improving the skills of facilitators might enable more appropriate support for practice teams during the Maturity Matrix session.

## Discussion

The model of facilitation enabled effective categorisation of the data extracted from the transcripts. The data suggest that whilst facilitators could effectively use skills associated with the first three areas, 'structuring the session', 'obtaining consensus' and 'handling disagreements', they were less able to use skills associated with the last area 'team learning'. Facilitators consistently and effectively used the basic skills required to administrate and manage the Maturity Matrix session (area 1). They were also able to effectively take the group through each of the eleven dimensions of the Maturity Matrix in turn, stimulating discussion amongst participants and steering them to a group decision about the current position of their practice (area 2). With respect to 'handling group dynamics', facilitators used skills effectively to handle resistance, deal with disagreements and ensure that all views were heard (area 3). However, the facilitators were less consistent in their ability to move the group from a position of discussing the assessment of organisational development in their practice to a position of discussing improvements that could be made (area 4). It might be expected that for every Maturity Matrix dimension where practices agreed that they were not at the highest level of development, there was potential to improve. However those conversations did not always automatically take place, possibly as a result of the wider context within which the Maturity Matrix session was held.

### Limitations

There are a number of weaknesses associated with this study. Firstly, out of necessity the study is based on findings using a convenience sample of practices. In determining the sampling strategy, it would have been better to consider further specific factors such as workload, teaching status and research status that might have had an impact on the Maturity Matrix profile. Secondly, participating practices were already taking part in a wider project as part of the European Practice Assessment collaboration. The Maturity Matrix session took place on the same day as the assessment of the practice using the European Practice Assessment tool, thus practices may have felt pressured and overburdened from a data collection perspective. Another weakness of this study was that the data came from only two facilitators and this restricts the generalisability of the findings. Finally, it was not possible to track the practices' development longitudinally over time and therefore, longer-term changes made as a result of using the Maturity Matrix were not identified. However, this study provides a basis for a future study using a larger sample of facilitators and practices with a wider range of characteristics.

### Findings in the context of existing literature

The existing literature on quality improvement in primary care suggests that instruments and methods are designed to achieve improvements either by using externally-led assessment or by encouraging practice-led learning [11,28-31]. It has also been suggested that externally-led assessments such as those exemplified by professionally-led accreditation systems dominate the quality improvement landscape [9]. In those primary care systems where top-down approaches exist, practice-led approaches are to be encouraged [32]. The existing literature on practice visiting has not explored the skills required for facilitating as opposed to assessing and educating. Facilitation is a style of interaction that encourages a group to solve its own problems. This study advances knowledge by developing a model of facilitation that supports quality improvement in practice teams who are distinct from other health care teams because, the doctors are mostly self employed and some of the practice team are employed whilst some are 'attached' (from other employers). For these reasons, it is difficult to rely on theories of organisational change that identify strategies for change that are top-down or mechanistic such as system theories.

### Implications for policy, practice and research

The pressure to externally assess family practices can stifle practice-led improvements [33,34]. There is scope to develop approaches such as the Maturity Matrix and similar as methods to stimulate practice-led improvements. The calls to integrate practice-led assessments with existing externally-led assessments imply that we need to understand more about how these methods affect the role of those who visit the practices either as assessors, educators or facilitators. The skills required by these roles are different. In addition, it may fall to one person as a practice visitor to switch between roles if practice-led and externally-led assessments become more closely integrated. The facilitator skills revealed in this study suggests that this role varies from that of a practice visitor as an assessor or educator as shown in Table 2. Furthermore, by mapping the roles of a practice visitor onto four major theories of organisational change [10] as shown in Table 2, a fourth role, that of a practice visitor stimulating improvements based on complexity theory is suggested. This is also an under researched area that is of increasing interest to researchers and policy makers alike [35].

**Table 2:** The variety of styles adopted by practice visitors

**Practice Visitor as:**	**Assessor**	**Expert/Educator**	**Facilitator**	**Interpreter**
Whose role is:	External assessment against standards.	Knowledge and skill transfer.	Consensus building to achieve group view.	Helping practice teams decide what and how they would like to improve.
Based upon:	Historical competence.	Best practice.	A template for process.	Practice based issues.
Controlling:	Outcome.	Outcome and process.	Process.	Neither outcome nor process, simply participation.
Theoretical basis:	Systems.	Systems and organisational development.	Organisational development.	Complexity.
Examples:	Accreditation systems.	Outreach project to set up systems for chronic disease management.	Maturity Matrix.	Action research.

To enable a practice team to plan practice-led improvements, facilitators need targeted training to skilfully move the practice beyond discussions about the assessment of current progress to discussions about improvement. Allowing adequate time for discussion and encouraging practice teams to discuss the organisational development with each other seem to be areas where improved facilitator skills can enable team learning to occur. However, the time allowed for the session may also be an issue; can eleven dimensions be adequately discussed in one to one and a half hours? Given that the facilitators were experienced and competent, it is possible that allowing more time for the sessions may have enabled facilitators to move the groups more effectively towards the discussion of improvements.

Future research into the Maturity Matrix should explore the impact on facilitator skills of increasing the time allowed for the meeting or reducing the number of dimensions considered. It should also explore what other facilitator skills could be added to the model to reflect facilitator knowledge about the timing of the use of particular skills as this issue was only considered briefly within this study. Finally, future studies should include an increased number of facilitators.

## Conclusion

Little research has been conducted into understanding the role of facilitators in using quality improvement instruments with general practice teams. This study proposes a model of facilitation that comprises four areas 'structuring the session', 'obtaining consensus', 'handling group dynamics' and 'enabling team learning'. Facilitators are more effective at displaying skills associated with the first three areas. This may partly be a function of training and also of the time allowed for the Maturity Matrix session. In addition, the model itself should be refined to capture relationships between the areas, particularly with respect to timing of the use of skills as this partly determines whether facilitation input is more or less effective. Quality improvement instruments such as the Maturity Matrix enable practice teams to take ownership of planning organisational development. However, their effectiveness is mediated by the extent to which facilitators employ both basic and higher level skills. This study suggests that facilitators need careful preparation for their role and practices need protected time in order to make best use of practice-led quality improvement instruments.

## Competing interests

The author(s) declare that they have no competing interests.

## Authors' contributions

MR was the lead researcher and undertook analysis of the transcripts in relation to the model developed.

AE was a supervisor and co-author and reviewed transcripts

RG was the lead supervisor for the study.

MM was a supervisor and co-author and provided resources to support the facilitators in their work.

GE was a co-author and reviewed transcripts

All authors read and approved the final transcript

## Pre-publication history

The pre-publication history for this paper can be accessed here:


